# The infectious diseases impact statement: a mechanism for addressing emerging diseases.

**DOI:** 10.3201/eid0202.960204

**Published:** 1996

**Authors:** E McSweegan

**Affiliations:** National Institute of Allergy and Infectious Diseases, National Institutes of Health, Bethesda, Maryland 20892-7630, USA. em8p@nih.gov

## Abstract

The use of an Infectious Diseases Impact Statement (IDIS) is proposed for predictive assessments of local changes in infectious diseases arising from human-engineered activities. IDIS is intended to be analogous to an Environmental Impact Statement. The drafting of an IDIS for specific activities, particularly in developing nations, would provide a formal mechanism for examining potential changes in local health conditions, including infected and susceptible populations, diseases likely to fluctuate in response to development, existing control measures, and vectors likely to be affected by human activities. The resulting survey data could provide a rational basis and direction for development, surveillance, and prevention measures. An IDIS process that balances environmental alterations, local human health, and economic growth could substantially alter the nature of international development efforts and infectious disease outbreaks.


### The Infectious Diseases Impact Statement: A Mechanism for Addressing Emerging Diseases

**Edward McSweegan, Ph.D.**
National Institute of Allergy and Infectious Diseases, National Institutes of Health, Bethesda, Maryland, USA

The use of an Infectious Diseases Impact Statement (IDIS) is proposed for predictive assessments of local changes in infectious diseases arising from human-engineered activities. IDIS is intended to be analogous to an Environmental Impact Statement. The drafting of an IDIS for specific activities, particularly in developing nations, would provide a formal mechanism for examining potential changes in local health conditions, including infected and susceptible populations, diseases likely to fluctuate in response to development, existing control measures, and vectors likely to be affected by human activities. The resulting survey data could provide a rational basis and direction for development, surveillance, and prevention measures. An IDIS process that balances environmental alterations, local human health, and economic growth could substantially alter the nature of international development efforts and infectious disease outbreaks.

A 1995 report by Aksoy et al. (1) describing the GAP (Turkish acronym for the Southeastern Anatolia Irrigation Project) irrigation project in Turkey suggests that anticipating the emergence or expansion of vector-borne and zoonotic diseases in a limited environment is a useful exercise. According to the report, a number of diseases (e.g., leishmaniasis, malaria, and schistosomiasis) are likely to increase in direct response to the expansion of irrigation and the increases in under water acreage and human population in the GAP region. The succinct overview of the disease and vector conditions in the GAP area could serve as a starting point for creating what will be referred to in this article as an Infectious Diseases Impact Statement (IDIS), a document that would be analogous to the Environmental Impact Statement (EIS) routinely used in the United States to assess the likely effects of construction, irrigation, agriculture, and similar activities on a local environment or region. An IDIS, however, would not assess the environment directly, but rather would predict changes in local disease patterns resulting from changes to the local environment.

Like an EIS, an IDIS would be a predictive and proactive assessment. Drafting an IDIS for a particular region or microenvironment would provide a formal mechanism for asking (and attempting to answer) specific questions about future changes in local health conditions. For example, what are the diseases and vectors in the given area? How are the proposed changes to the environment (e.g., dam-building, forest-clearing) likely to change the incidence and the prevalence of those diseases and vectors? What actions should be taken during the course of a given project and in the future to prevent potential increases in disease and vector populations? If an increase in human disease is likely, is the expense of the proposed project warranted? Will the economic benefits of a particular development or agriculture project be offset by increased costs in health care, vaccination, and vector control?

The 1969 National Environmental Policy Act was designed to provide a legal mechanism in the United States for evaluating potential impact to the environment from development activities and for permitting the public to participate in the evaluation process at the earliest stages (i.e., "scoping"). A Council of Environmental Quality in the executive branch of the federal government was established as a monitor, and the EIS process was implemented to inform decision makers and the public of potential environmental problems and reasonable alternatives to proposed actions. Environmental Protection Agency (EPA) requirements for environmental assessments are outlined in the Code of Federal Regulations (CFR). An EIS is intended to prospectively examine impact "upon the quality of the human environment of the United States and, in appropriate cases, upon the

Address for correspondence: Edward McSweegan, Ph.D., National Institute of Allergy and Infectious Diseases, National Institutes of Health, Solar Bldg., Rm. 3A34, Bethesda, MD 20892-7630, USA; fax: 301-402-0659; e-mail: em8p@nih.gov.

environment of the global commons outside the jurisdiction of any nation" (46 CFR sec. 504.7). The requirements of an EIS typically include descriptions of human populations in the designated area, current land use patterns, air quality, noise levels, locations of wetlands and coastal zones, sites of historical or cultural value, and non-point source pollution. The protection of human health is implied in the EIS process, but this concern is usually assumed to pertain to the location of industrial plants and dumps and to exposure to toxic chemicals, heavy metals, ionizing radiation, and pesticides. The EIS process contains no explicit reference to infectious diseases or disease vectors affecting human health in response to deliberate environmental changes. In 1995, the only published EIS references to diseases, infectious or otherwise, were for proposed control measures at two California plant nurseries. Yet past events suggest that attention should be directed toward changes in infectious disease patterns directly attributable to human-engineered events.

For example, the construction of the Aswan Dam in Egypt is widely believed to have precipitated the appearance of Rift Valley fever (RVF) in Egypt during the 1970s (2). Tens of thousands of RVF cases and hundreds of deaths followed. Similarly, completion of the Diama Dam in Senegal, in 1987, led to epidemics of malaria and RVF (3); impoundment of the Volta Lake in Ghana, in 1968, led to an explosive outbreak of schistosomiasis (4). Increased agriculture on the Argentine pampas and along the edges of Bolivian forests has contributed to frequent hemorrhagic fever outbreaks caused by Junin and Machupo viruses, respectively (5). Mining operations in the Brazilian jungles have led to outbreaks of malaria (6). Road-building projects under way in Papua New Guinea are likely to bring large numbers of susceptible human hosts into contact with rare and yet-to-be-discovered viruses. These epidemics and encounters with new diseases are the unforeseen consequences of critically altering the local environment. As a consequence, development and agriculture projects initiated to improve the lives of local populations can have the opposite effect by increasing disease prevalence and causing new epidemics. Embedding an IDIS requirement into the planning and execution of large-scale projects likely to alter local environments could prevent new epidemics and reduce infectious disease–associated morbidity and mortality.

### How Would an IDIS Work?

An IDIS would first need to be established as an integral component of any activity likely to affect the health of a local population. In tropical and developing regions of the world, that would include a variety of national and international development activities. The area designated for large-scale alteration would be surveyed for current disease vectors, and the local populations would be examined for diseases likely to be affected by the project in question. The quality of the surveillance and the extrapolation of expected changes brought on by a particular activity would vary, depending on the knowledge of diseases, vectors, local host immunity, and other factors. Although the variables increase the margin of uncertainty in such extrapolations, these estimates would be expected to improve as the state of field and laboratory research improves and experience with preparing an IDIS increases. A retrospective examination of earlier projects in similar environments would also provide information for developing an IDIS. The standards of "existing credible scientific evidence" and "reasonably foreseeable" impact that current environmental impact assessments rely on could also be applied to the early stages of the IDIS process.

The resulting preproject assessment would provide a snapshot of conditions in a defined area, including the following: diseases likely to fluctuate in response to project activities, numbers of infected and susceptible hosts, existing control measures, and vectors likely to be affected by project activities. Such baseline data are frequently absent from development and agriculture activities (7). Knowing what diseases are already present, and how they might be changed, allows one to ask how anticipated changes in disease prevalence and distribution might be prevented or controlled through changes in the proposed project, improved case finding and treatment, changes in local sanitation and housing, increased vaccination or prophylaxis, or pest management programs. Some or all of the above health maintenance measures could then become components of the overall planning, budgeting, and execution of any major development or agricultural activity in the area. Health and health maintenance would become factors in the overall design and cost of the project. In many instances, local disease surveillance would become an ongoing part of the project, with supplemental assessments being made to refine the original IDIS.

### Who Would Request an IDIS, and Who Would Respond to the Request?

Initial candidates would likely be donor organizations (the U.S. Agency for International Development and the World Bank, for example) that provide funding and oversight. In the absence of federal or international statutes, these organizations have the stature and financial capability to make infectious disease control an integral part of their development projects. Indeed, they should have an urgent interest in doing so because increases in diseases or new epidemics increase financial demands on them for medicines, vaccines, and pest control. In the end, more money would be spent beating back the outbreaks and epidemics that foresight might have prevented. National health ministries, state and territorial health departments, and local medical communities in developing countries might also request or initiate an IDIS. The practice of drafting an IDIS and implementing its recommendations might also rejuvenate underfunded areas of international health, vector biology, parasitology, and medical entomology as professionals in these fields are called on to conduct infectious disease assessments of development activities. The peer-reviewed literature and electronic services such as ProMED, Outbreak, and the World Health Organization (WHO) and Centers for Disease Control and Prevention World-Wide Web sites could provide the public "scoping" role that posting in the *Federal Register* and allowing a period for public comment provide in the EIS process in the United States.

The first application of an IDIS to a large-scale development or environmental activity could come from western donor organizations working in the developing world. The successful demonstration of an IDIS could encourage other organizations, national health officials, and health activists to push for the routine integration of public health with national development. This could happen in the United States, as well. The United States recently experienced the emergence of Sin Nombre virus in the Southwest and is theoretically open to the introduction of five vector-borne diseases: malaria, Rift Valley fever, yellow fever, dengue, and arbovirus encephalitides (15). Public health officials and citizen activists could initiate independent IDIS for activities perceived to threaten the balance between health, the environment, and domestic productivity.

### What are the Strengths and Limitations of the IDIS Process?

A project-embedded IDIS would not be the same as an environmental management program, which seeks to control disease vectors through environmental modification and manipulation and through reduced human contact with vectors (8). An IDIS would, in fact, precede environmental management control measures by first postulating the likely emergence of specific pathogens and vectors. The usefulness of an IDIS lies in its ability to provide a conceptual framework for identifying potential disease problems, and, indeed, preventing them by altering or curtailing the very activities that could lead to disease emergence.

In an activist sense, an IDIS could be wielded as a tool of caution or prevention, much as an EIS is wielded in the United States to alter or halt some activity perceived to be a threat to the environment. That ability to influence potential changes and to affect health could be vital; public health concerns connected with agricultural and developmental projects are usually a low priority among foreign ministries, international donor organizations, and engineers (9); neglecting them can leave the full benefits of development unrealized.

Lest anyone imagines that an IDIS could be used solely as a tool of the political Left, as a kind of "liberation microbiology," it is important to point out that the same IDIS could be used to justify the use of pesticides and other organized control measures, including the relocation of local populations. Recently, for example, pesticide use has come under attack by various environmental groups, and donor organizations have become increasingly reluctant to fund such activities (10). In the United States, EPA's Endangered Species Act has also tended to thwart the use of pesticides because of potentially adverse impact on some birds and mammals (11). However, an IDIS describing the probable emergence of important disease vectors could be used to justify such use. Thus, a health care issue could be twisted into a health scare by either the political Right or the Left. The recent ratification of the North American Free Trade Agreement (NAFTA) was preceded in the United States by an effort to stall the treaty with an EIS requirement. If an IDIS had predicted new disease outbreaks from increased border trade and traffic, that concern might have had greater impact on the public imagination than more abstract concerns about atmospheric particulates in the border region and could have been effectively used by anti-NAFTA forces. An IDIS should be not a political tool but rather a valuable information source that helps guide economic development and land use.

### How Can an IDIS Complement Existing Surveillance Systems?

Almost half of the planet's five billion people are at risk for one or more vector-borne diseases (12, 13). Surveillance remains a key tool for monitoring these diseases and identifying new cases and outbreaks. Four types of surveillance are used in the control of vector-borne diseases: 1) recording human cases, 2) determining vector distribution and infectivity, 3) monitoring vertebrate reservoirs, and 4) tracking weather patterns to predict vector distribution (14). But throughout the developing world and across tropical boundaries, effective and continuous surveillance is extremely difficult, if not impossible. Cases are missed; outbreaks go unreported. Effective case reporting and continuous field monitoring are best conducted in limited, well-defined areas. Within the microenvironments of human activities, an IDIS could provide valuable baseline surveillance data before changes to that area occur and affect disease and vector distributions. This information could provide a rational basis and direction for ongoing monitoring and corrective measures (e.g., vaccination, relocation, pest control). Focusing on a limited area and a limited number of diseases in that area may also expand the use of promising but underutilized technologic methods such as remote sensing and geographic information systems (GIS).

Haines et al. (15) noted the importance of vector-borne disease monitoring and recommended that remote sensing and GIS be used to detect changes in ecosystems and vector populations. To a large extent, however, the advantages of satellite imagery and GIS have not been realized, in part, because of the frequent absence of "ground truth" (data on diseases, vectors, and other factors in the area) and of having to wait to observe natural environmental changes likely to affect disease and disease transmission (16-18). Satellite imagery for much of the planet has been collecting in databases since 1972 (16). By 1998, accumulated satellite data will be in the petabyte (1,000 terabyte) range, 1,000 times larger than the contents of the Library of Congress (B. Montgomery, NASA, pers. comm.). High-resolution, multispectral, multiyear images for many potential development and agricultural sites are available. Using a preproject IDIS to "ground truth" the project's environment with current satellite imagery, it may be possible to more completely describe local disease and vector conditions and make more accurate predictions about their plasticity during periods of construction, flooding, or farming. The result would be a firmer linkage of ground surveillance and satellite imagery to monitor public health changes within a well-defined and limited environment.

In recent years, the sudden emergence of rare or forgotten diseases such as Ebola virus infection, dengue, yellow fever, plague, and hantavirus (Sin Nombre virus) infection has attracted the attention of the public and inspired renewed commitments to surveillance and control. WHO recently formed a rapid response unit (the Division of Emerging, Viral and Bacterial Diseases Surveillance and Control) to deal with outbreaks of new and reemerging infections (19). Similarly, nine Southeast Asian countries held a meeting on emerging diseases and concluded that each country should also develop rapid response teams for epidemics (20). However, these disease control efforts are almost entirely passive, with staff, equipment, and budgets idling in anticipation of something eventually happening somewhere. It is difficult to maintain a high degree of public and financial support for such wait-and-see approaches to disease control. The United States has suffered a serious decline in national surveillance and outbreak investigations, in part, because of decreased support for passive monitoring programs (11).

### Is an IDIS Really Needed When the Existing EIS Statutes Already Cover Human Health and Safety Concerns?

In the United States the need is not clear. Infectious diseases caused by environmental manipulation may be assumed to fall under the general EIS category of human health. However, infectious diseases have not often been considered in the past, and it is easy to imagine that if they were a factor in the EIS process, an environmental/infectious disease issue could be smothered under the weight of government regulations and the adversarial legal system. EPA operates under 16 federal statutes and 70 congressional committees and subcommittees and is engaged in some 600 lawsuits at any given time (21). Moreover, emerging infectious disease issues could bring EPA and the EIS process into conflict with the missions of federal agencies and state and local health departments. Outside the United States, beyond federal statutes and informed public debate, the need for an IDIS is clearer. In the developing world, epidemics and substandard health care are common, and the national goals of healthy environment and healthy economy are usually at odds. An IDIS process that balances environmental alterations, local human health, and economic development could substantially alter the nature of international development efforts and infectious disease outbreaks.

To the ancient Greeks, the past appeared in front of them, real and visible; the future was behind them, unseen and unknowable. With that perspective, they were always glancing nervously backward, looking for a future that usually managed to creep up and tap them on the shoulder. In a sense, we have the same perspective for disease surveillance and control that the ancient Greeks had for time. Past epidemics and our responses to them are readily apparent; it is that unexpected tap on the shoulder by a hantavirus or an Ebola virus that is always so startling. We cannot know when and where such pathogens will emerge. Their appearance is often a chance event initiated by unpredictable changes in weather or the accidental encounter of a single person with a mysterious vector. These taps on the shoulder are an affront to our sense of control and understanding of disease. Moreover, it is unsettling to the public's sense of security and its faith in medical research. Although we cannot expect to eliminate the surprises of emergent pathogens in the near future, we can take control of situations in which our own actions directly lead to the emergence of diseases. Generating an IDIS in areas where human activities are likely to disrupt endemic-disease patterns would be an important step in controlling future outbreaks. Routine application of a preproject IDIS could improve local surveillance and health care planning by 1) providing baseline data on endemic-disease and vector prevalence and competence; 2) embedding projected health maintenance costs into the planning and cost of any project or activity likely to influence the environment and public health; and 3) providing a mechanism for instituting project alterations and health care measures to offset adverse effects on the health of local populations.

### Acknowledgments

Special thanks to Dr. Michael Gottlieb, National Institute of Allergy and Infectious Diseases, Parasitology and International Programs Branch, and Donald C. Baur, Esq., Perkins-Cole, Washington, D.C. for critical comments and suggestions.

*Dr. McSweegan is a member of the Parasitology and International Programs Branch at the National Institute of Allergy and Infectious Diseases (NIAID), National Institutes of Health, Bethesda, Md. He has a long-standing interest in international science and health, and emerging diseases. He served in the U.S. State Department as an AAAS Fellow before joining NIAID's Tropical Medicine and International Research Office in 1988.*

### References

1. Aksoy S, Ariturk S, Armstrong MYK, Chang KP, Dörtbudak Z, Gottlieb M, et al. The GAP project in southeastern Turkey: the potential for emergence of diseases. Emerging Infectious Diseases 1995; 1:62-3.
2. Meegan JM, Shope RE. Emerging concepts on Rift Valley fever. In: Pollard M, editor. Perspectives in virology. New York: Alan R. Liss, 1981.
3. Lederberg J, Shope RE, Oaks SC, editors. Emerging infections: microbial threats to health in the United States. Washington, DC: Institute of Medicine, National Academy Press, 1992;71-2.
4. Scott D, Senker K, England EC. Epidemiology of human *Schistosoma haematobium* infection around Volta Lake, Ghana, 1973-75. Bull WHO 1982;60:89-100.
5. Morse SS. Emerging viruses: defining the rules for viral traffic. Perspect Biol Med 1991;34:387-409.
6. de Andrade ALSS, et al. High prevalence of asymptomatic malaria in gold mining areas in Brazil. Clin Infect Dis 1995;20:475.
7. Service MW. Rice, a challenge to health. Parasitol Today 1989;5:162-5.
8. Ault SK. Environmental management: a re-emerging vector control strategy. Am J Trop Med Hyg 1994;50(Suppl):35-49.
9. Silver GA. 1995. International Health Organization Policy Watch. The Federation of American Scientists. (http://www.clark.net/pub/gen/fas/ihm).
10. Arata AA. Difficulties facing vector control in the 1990s. Am J Trop Med Hyg. 1994;50(Suppl):6-10.
11. Longstreth J, Wiseman J. Human health. In: Smith JB, Tirpak DA, editors. The potential effects of global climate change on the United States: Appendix G, Health. Washington, DC: U.S. Environmental Protection Agency, 1989.
12. Beck LR, Rodriguez MH, Dister SW, Rodriguez AD, Rejmankova E, Ulloa, et al. Remote sensing as a landscape epidemiologic tool to identify villages at high risk for malaria transmission. Am J Trop Med Hyg 1994;51:271-80.

13. Knudsen AB, Sloof R. Vector-borne disease problems in rapid urbanization: new approaches to vector control. Bull WHO 1992;70:1-6.
14. Consortium for International Earth Sciences Information Network (CIESIN) Thematic Guides. Provisional Release. 1995. Programs for surveillance, treatment, and control of vector-borne diseases.
15. Haines A, Epstein PR, McMichael AJ. Global health watch: monitoring impacts of environmental change. Lancet 1993;342:1464-9.
16. Washino RK, Wood RL. Application of remote sensing to arthropod vector surveillance and control. Am J Trop Med Hyg. 1994;50Suppl:134-44.
17. Rogers DJ, Williams BG. Monitoring trypanosomiasis in space and time. Parasitology 1993;106:S77-S92.
18. Barinaga M. Satellite data rocket disease control efforts into orbit. Science 1993; 261:31-2.
19. World Health Organization. WHO establishes new rapid-response unit to combat growing world-wide threat of emerging diseases. WHO/75. Press release, 17 October 1995.
20. Plianbangchang S. Southeast Asia intercountry consultative meeting. Emerging Infectious Diseases 1995;1:158.
21. Environmental Protection Agency. The common sense initiative. Pub. No. EPA100F94004. Washington, DC: Environmental Protection Agency.
